# Novel *Bartonella* Species in Insectivorous Bats, Northern China

**DOI:** 10.1371/journal.pone.0167915

**Published:** 2017-01-12

**Authors:** Hui-Ju Han, Hong-ling Wen, Li Zhao, Jian-wei Liu, Li-Mei Luo, Chuan-Min Zhou, Xiang-Rong Qin, Ye-Lei Zhu, Xue-Xing Zheng, Xue-Jie Yu

**Affiliations:** 1 Department of Epidemiology, School of Public Health, Shandong University, Jinan, Shandong, China; 2 Department of Microbiology, School of Public Health, Shandong University, Jinan, Shandong, China; 3 Shandong Center for Disease Control and Prevention, Jinan, Shandong, China; 4 University of Texas Medical Branch, Galveston, Texas, United States of America; University of Minnesota, UNITED STATES

## Abstract

*Bartonella* species are emerging human pathogens. Bats are known to carry diverse *Bartonella* species, some of which are capable of infecting humans. However, as the second largest mammalian group by a number of species, the role of bats as the reservoirs of *Bartonella* species is not fully explored, in term of their species diversity and worldwide distribution. China, especially Northern China, harbors a number of endemic insectivorous bat species; however, to our knowledge, there are not yet studies about *Bartonella* in bats in China. The aim of the study was to investigate the prevalence and genetic diversity of *Bartonella* species in bats in Northern China. *Bartonella* species were detected by PCR amplification of *gltA* gene in 25.2% (27/107) bats in Mengyin County, Shandong Province of China, including 1/3 *Rhinolophus ferrumequinum*, 2/10 *Rhinolophus pusillus*, 9/16 *Myotis fimbriatus*, 1/5 *Myotis ricketti*, 14/58 *Myotis pequinius*. Phylogenetic analysis showed that *Bartonella* species detected in bats in this study clustered into ten groups, and some might be novel *Bartonella* species. An association between *Bartonella* species and bat species was demonstrated and co-infection with different *Bartonella* species in a single bat was also observed. Our findings expanded our knowledge on the genetic diversity of *Bartonella* in bats, and shed light on the ecology of bat-borne *Bartonella* species.

## Introduction

*Bartonella* is the only genus in the family *Bartonellaceae* of order *Rhizobiales*. *Bartonella* are facultative intracellular bacteria that infect the erythrocytes and endothelial cells of a wide variety of mammals worldwide [1,2]. In humans, *Bartonella* species are the causative agents of Peruvian bartonellosis, cat scratch disease, trench fever, and bacillary angiomatosis [3], and *Bartonella* infection can also cause various cardiovascular, neurological, and rheumatologic diseases [4,5]. An Increasing number of *Bartonella* species are identified as human pathogens [6,7], and at least fifteen *Bartonella* species are associated with human infections [3,8].

Numerous studies demonstrated that bats and their ectoparasites carried diverse *Bartonella* species [9]. Bats were implicated as hosts for a human pathogen, *Bartonella mayotimonensis* [10]. However, the role of bats as the zoonotic sources of bartobellosis is unclear. *Bartonella* species in bats have been investigated in America, Europe, Africa and Asia [11–20], however, in Asia, *Bartonella* species in bats have only been reported in regions of South Asia [21,22], and no such relevant studies of *Bartonella* have been conducted in mainland China, the Northern of which harbors a d number of endemic insectivorous bat species. To expand our understanding of *Bartonella* species in bats, we investigated the prevalence and genetic diversity of *Bartonella* species in bats in Mengyin County, Shandong Province of China.

## Materials and Methods

### Ethics Statement

This study was carried out in accordance with the Guidelines of Regulations for the Administration of Laboratory Animals (Decree No. 2 of the State Science and Technology Commission of the People's Republic of China, 1988). The collection of bats for microbiological studies was approved by the Ethics Committee of Prevention Medicine of Shandong University (No.20150501), and all efforts were made to minimize discomfort to bats. Bats were sampled with the help of Mengyin County Center for Disease Control and Prevention. For sampling in karst cave and human houses, it was approved by the cave owner and house owners, respectively. While for sampling in cave and city sewer, no specific permission was needed for they were not private places. There are no endangered or protected species in the sampling habitats.

### Bat Collection

**Fig 1:**
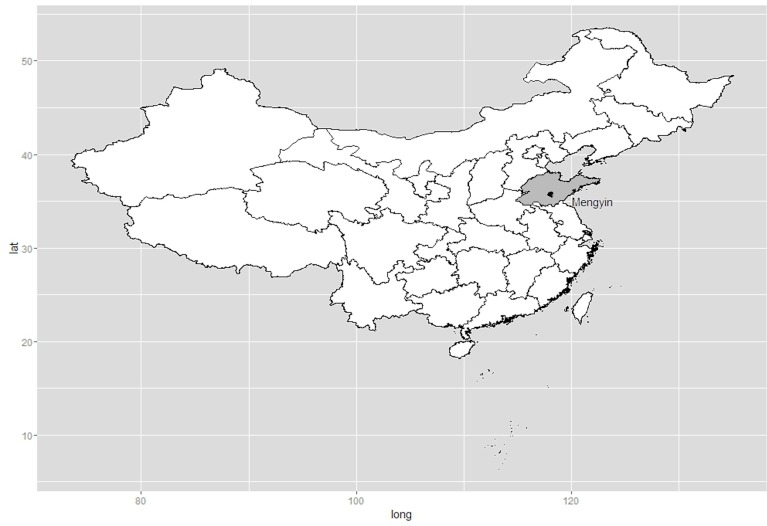
Geographic location of Mengyin County in Shandong, China. The map was constructed using R 3.3.2 software (https://www.r-project.org/).

Bats were collected from March to October, 2015, in Mengyin County (551,000 inhabitants), Shandong Province, which is located in the east of China (Fig 1).

Bats were captured using mist nets settled near the entrance of karst caves or sewers at sunset when bats left roosts for night feeding and were collected from the mist nets in the next early morning. Bats were also captured by hand with butterfly nets within caves and residential houses. Captured bats were anesthetized by an intramuscular injection of chloral hydrate. Blood samples were obtained immediately after sedation by puncturing into the median artery. The amount of blood sampled varied from 0.2–0.5 mL according to the size of bats. Blood samples were stored at 4°C for a few hours before centrifuged for 5 minutes at 4000 rpm to separate the blood clots and the serum. Then the bats were euthanized by injection of overdose chloral hydrate. Selected internal organs were collected for further microbiological analysis. All specimens were transported in liquid nitrogen and were then stored frozen at –80°C until use. Bat species was identified by sequencing PCR product of the cytochrome B (*cytB*) gene amplified from bat liver DNA as described previously [23].

### Amplification of *Bartonella gltA* Gene

DNA was extracted from blood clots with QIAGEN DNA kit (Qiagen, Valencia, CA) according to the manufacturer’s instructions. All 107 blood samples were initially screened for the RNA polymerase B (*rpoB*) gene of *Bartonella* species with primers BArBF/BArBR [24]. Samples with positive *rpoB* results were subsequently amplified for *Bartonella* citrate synthase gene (*gltA*), which was a reliable tool for distinguishing closely related *Bartonella* genotypes [25]. PCR amplifications of *gltA* gene was performed with primers BhCS781.p/ BhCS1137.n or CS443f/ CS1210, targeting a 379-bp and 767-bp fragment of *gltA* gene, respectively [26,27]. The PCR reaction was conducted in a 50-μL mixture containing 25 μL of DreamTaq Green PCR Master Mix (2X) (Thermo Scientific, Waltham, MA, USA), 0.8 μL of 25 μmol/L of each forward and reverse primer (Sangon Biotech, Shanghai, China), 16.4 μL of nuclease-free water, and 7 μL blood DNA of each sample. Nuclease-free water was used as a negative control, and no positive control was used. PCR was performed under the following conditions: 1 denaturing cycle at 95°C for 5 min followed by 40 cycles at 95°C for 30 s, 51°C for 40 s (BhCS781.p/ BhCS1137.n) or 48°C for 40 s (CS443f/ CS1210r), and 72°C for 90 s, and an additional final cycle at 72°C for 10 min. Products were analyzed by 1.2% agarose gel electrophoresis and detected by using ethidium bromide under UV light. PCR products with expected size (379-bp for BhCS781.p/ BhCS1137.n and 767-bp for CS443f/ CS1210) were excised from gels and extracted using a Gel Extraction Kit (Promega, Madison,WI), and were then cloned into pMD 19-T vectors (TaKaRa, Shiga, Japan), all according to the manufacturer’s instructions. Three recombined plasmid for each PCR product were sequenced on both strands.

### Phylogenetic Analysis

Sequence chromatograms and sequence analysis were examined by using Chromas (Technelysium, Tewantin, QLD) and BLAST program (http://blast.ncbi.nlm.nih.gov/Blast.cgi), respectively. *Bartonella gltA* sequences were aligned and trimmed with MEGA7.0 to a 327-bp fragment (positions 801–1127), which was commonly used for taxonomic classification of *Bartonella* species[25]. The phylogenetic tree was constructed with the neighbor-joining method by using MEGA7.0, and bootstrap values were calculated with 1,000 replicates.

## Results

### Bat Collection

A total of 145 insectivorous bats were collected from Mengyin County, Shandong Province of China. Bats were captured from 4 different habitats and were molecularly identified into 6 species. *Rhinolophus ferrumequinum* (Greater horseshoe bat) and *Rhinolophus pusillus*(Least horseshoe bat) were collected from a karst cave, while *Myotis fimbriatus* (Fringed long-footed bat) and *Myotis ricketti* (Rickett's big-footed bat) were captured from a city sewer. *Eptesicus serotinus* (Common serotinus bat) were collected from two farmers’ houses and *Myotis pequinius* (Beijing mouse-eared bat) were collected from a cave. Blood samples were obtained from 107 living bats and were used in this study. Blood were not obtained from 38 bats which were died before collecting blood or were difficult to collect blood because of small body size.

### Prevalence of *Bartonella* Species in Bats

Of 107 bats tested, *Bartonella gltA* gene was successfully amplified and sequenced from 27(25.2%) bats. Prevalence of *Bartonella* species by *gltA* in bats was as follows (Table 1): *Rhinolophus ferrumequinum*, 1/3 (33.3%); *Rhinolophus pusillus*, 2/10 (20.0%); *Eptesicus serotinus*, 0/15 (0.00%); *Myotis fimbriatus*, 9/16 (56.3%); *Myotis ricketti*, 1/5 (20.0%); and *Myotis pequinius*, 14/58 (24.1%).

**Table 1:** Prevalence of *Bartonella* among bats in Mengyin County, China.

Bat Species	No. of bats	Positive rate (%)	*Bartonella* phylogroup (Positive No.)
*Rhinolophus ferrumequinum*	3	33.3	VII(1)
*Rhinolophus pusillus*	10	20.0	IV(1); VIII(1)
*Eptesicus serotinus*	15	0	
*Myotis fimbriatus*	16	56.3	III (4)^a^; VI(3); IX(2)^a^; X(1)
*Myotis ricketti*	5	20.0	Ⅵ(1)
*Myotis pequinius*	58	24.1	Ⅰ(1); II(5)^b^;Ⅴ(2); VI(5)^b^; IX(1); X(1)
**Total**	**107**	**25.2**	

^a^ One *Myotis fimbriatus* was co-infected with phylogroup III and IX.

^b^ One *Myotis pequinius* was co-infected with phylogroup II and VI.

### Genetic Diversity and Sequence Clustering of *Bartonella* Species

**Fig 2:**
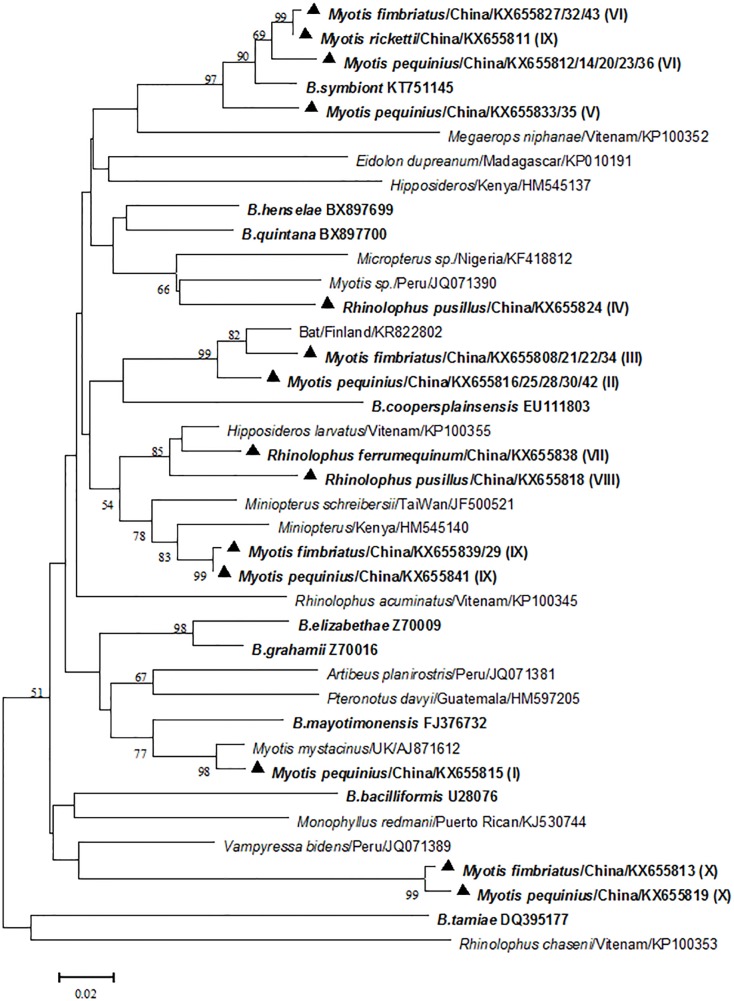
Phylogenetic relationships of the bat-borne *Bartonella* species based on the 327-bp fragement of *gltA* gene. *Bartonella* detected in bats were provided with the Latin name of the bat genus, the sampling site and the GenBank accession number. *Bartonella* detected in this study was shown in boldface and marked with a triangle and the phylogroups was provided after the GenBank accession number in parentheses (I–X). Reference *Bartonella* species were also shown in boldface.

Twenty-nine *gltA* sequences of *Bartonella* species were obtained from 27 bats, with 1 sequence from each bat for 25 bats and 2 divergent sequences from each bat for 2 bats. Subsequent analyses of the 29 *gltA* sequences revealed that they clustered into 10 phylogroups (I–X) with 4.4%-19.9% divergence (Fig 2). Five phylogroups (II, III, IV,VIII, X) showed <96% homology to all publicly available *Bartonella gltA* sequences in GenBank, and phylogroup X, in particular, shared only 84% sequence similarity with the most closely related *Bartonella* species (GenBank accession number JQ071389), indicating they might represent novel *Bartonella* species.

### Host-Specificity of *Bartonella* Species in Bat Species

Based on their *gltA* identity, our investigation showed an association between specific *Bartonella* genotypes and bat species, despite that some *Bartonella* species can infect several bat species (Table 1). PhylogroupⅠ, II and V were only detected in *Myotis pequinius*, phylogroup III in *Myotis fimbriatus*, phylogroup IV and VIII in *Rhinolophus pusillus*, phylogroup VII in *Rhinolophus ferrumequinum*; while phylogroup VI were detected in *Myotis pequinius*, *Myotis fimbriatus* and *Myotis ricketti*; phylogroup IX and X were detected in *Myotis pequinius*, *Myotis fimbriatus*. In addition, co-infection with different *Bartonella* species was observed, one *Myotis fimbriatus* was co-infected with phylogroup II and VI, and one *Myotis pequinius* with phylogroupⅠand IX. The *cytB* gene representing the 6 bat species and the 29 *gltA* sequences were deposited in GenBank with accession number from KX655808 to KX655843.

## Discussion

We analyzed the prevalence and genetic diversity of *Bartonella* species in bats in mainland China. Five out of 6 bat species investigated was positive to *Bartonella* species with extremely high prevalence rates ranging from 20.0%-56.3%, with the highest detection rate in *Myotis fimbriatus*. Phylogenetic analyses of the *Bartonella gltA* sequences indicated that bats in Mengyin County harbored diverse *Bartonella* species that could be clustered into 10 phylogroups, 5 strains appeared to be novel *Bartonella* species. One bat species was negative for *Bartonella* species; however, it is improper to speculate that it was free from *Bartonella* infection because of the limited sample size of this bat species.

Our investigation showed that an association existed between specific *Bartonella* genotypes and bat species, despite that some *Bartonella* genotypes could infect several bat species. A previous study also indicated a definite host-specificity for *Bartonella* species in bat species [15]. A recent study indicated that divergent adaptation was of less importance than codivergence in the formation of *Bartonella* species in bats, supporting the primacy of adaptation to bat hosts in the evolution of bat-associated *Bartonella* species [9]. Co-infection of different *Bartonella* species in a single bat was also observed and this might have an important implication on the generation of genetically diverse *Bartonella* through recombination, which have been observed in rodent-borne *Bartonella* [24].

*Bartonella* are considered as vector-transmitted agents, and diverse arthropods, such as sandflies, lice, fleas, ticks, and mites, have been implicated as potential vectors [28]. Numerous studies of *Bartonella* in bat ectoparasites showed positive results [12,13,17,19,29–34]. Ectoparasites may play an important role in the maintenance and transmission of *Bartonella* among bats [10,13,35]. In consideration of the special characteristics of bats, such as long lifespan, social animals in large number, diverse species, worldwide distribution [36], bats might be important reservoirs for *Bartonella* species.

Bats are considered as reservoirs of many viruses, and spillover of some bat-borne viruses to human may lead to severe human diseases [36]. A study showed that bats were reservoirs for human pathogenic *Bartonella mayotimonensis* [10], suggesting the spillover of bat-borne *Bartonella* species to humans. However, a prevalence study conducted in Ghana found no evidence of bat-associated *Bartonella* infection in humans, suggesting that the species isolated from *E*. *helvum* bats never or rarely infects humans in Ghana [37]. So far, the pathogenesis of most bat-borne *Bartonella* species to humans remains unknown, and further studies are needed to clarify the zoonotic potential of bat-borne *Bartonella* species.

## Conclusion

Our study provided the pioneer report of the genetic diversity of *Bartonella* species in bats in Mengyin County of Shandong Province, China. *Bartonella* species were detected by PCR amplification of *gltA* gene in 25.2% (27/107) of bats. Phylogenetic analysis showed that *Bartonella* species detected in bats clustered into several genotypes, with diverse potentially novel *Bartonella* species. Some *Bartonella* species are specifically associated with special bat species, and co-infection with different *Bartonella* genotypes in a single bat was also observed. Our findings expanded our knowledge on the genetic diversity of *Bartonella* infection in bats, and shed light on the ecology of bat-borne *Bartonella* species.
